# MutT-related proteins are novel progression and prognostic markers for colorectal cancer

**DOI:** 10.18632/oncotarget.22393

**Published:** 2017-11-11

**Authors:** Jin Li, Cheng-Cheng Yang, Xin-Yuan Tian, Yun-Xuan Li, Ju Cui, Zhe Chen, Zhou-Lu Deng, Fu-Jun Chen, Hiroshi Hayakawa, Mutsuo Sekiguchi, Jian-Ping Cai

**Affiliations:** ^1^ Peking University Fifth School of Clinical Medicine, Beijing Hospital, Beijing, P.R. China; ^2^ School of Pharmacy, Wenzhou Medical University, Wenzhou, P.R. China; ^3^ The MOH Key Laboratory of Geriatrics, Beijing Hospital, National Center of Gerontology, Beijing, P.R. China; ^4^ Department of General Surgery, China-Japan Friendship Hospital, Beijing, P.R. China; ^5^ Department of Anorectal Surgery, First Affiliated Hospital of Jiamusi University, Jiamusi, P.R. China; ^6^ Frontier Research Center, Fukuoka Dental College, Fukuoka, Japan

**Keywords:** MutT-related proteins, oxidized nucleotides, colorectal cancer, clinical relevance, prognosis

## Abstract

**Background:**

MutT-related proteins, including MTH1, MTH2, MTH3 and NUDT5, can effectively degrade 8-oxoGua-containing nucleotides. The MTH1 expression is elevated in many types of human tumors and MTH1 overexpression correlates with the tumor pathological stage and poor prognosis. However, the expression of other MutT-related proteins in human cancers remains unknown. The present study systematically investigated the expression of MTH1, MTH2, MTH3 and NUDT5 in human colorectal cancer to establish its clinical significance.

**Methods:**

Amounts of *MutT-*related mRNA and protein in CRC cell lines were assessed by qRT-PCR and Western blotting, respectively. Furthermore, the MutT-related protein expression was evaluated by immunohistochemical staining of tissue microarrays containing 87 paired CRC tissues and by Western blotting of 44 CRC tissue samples. Finally, the effect of knockdown of MutT-related proteins on CRC cell proliferation was investigated.

**Results:**

The expression of MTH1, MTH2, MTH3 and NUDT5 was significantly higher in CRC cells and CRC tissues than normal cells and tissues, and this phenomenon was significantly associated with AJCC stage and lymph node metastasis of CRC specimens. CRC patients with high expression of MTH1, MTH2 or NUDT5 had an extremely poor overall survival after surgical resection. Notably, NUDT5 was an independent prognostic factor of CRC patients. We found that knockdown of MutT-related proteins inhibited CRC cell proliferation.

**Conclusions:**

We showed for the first time that MutT-related proteins play an important role in CRC progression and prognosis. Further investigations are needed to elucidate the role of these proteins in CRC progression and their potential use for therapeutic targets.

## INTRODUCTION

A variety of reactive oxygen species (ROS) are generated in living cells during normal cellular metabolism, and their production is further enhanced by exposure to exogenous chemicals and ionizing radiation [1]. The formation of ROS leads to the oxidation of cellular components, including proteins, nucleic acids, carbohydrates and lipids, and disturbs their normal functions. DNA and RNA precursor nucleotides are also subjected to oxidative damage. Since guanine has the lowest oxidation potential, it is most readily oxidized to form 8-oxo-7,8-dihydroguanine (8-oxoguanine, 8-oxoG) [2]. 8-oxoGua-containing nucleotides, such as 8-oxoGDP, 8-oxoGTP, 8-oxo-dGDP, 8-oxo-dGTP, are formed in the nucleotide pool [3], and can be incorporated into DNA as well as RNA. Since 8-oxoG can pair with adenine and cytosine at almost equal efficiencies, it can cause both replicational and transcriptional errors [4–6]. Therefore, 8-oxoGua-containing nucleotides are potentially hazardous for genetic stability.

The MutT protein of *Escherichia coli* is capable of hydrolyzing a wide range of 8-oxoGua-containing nucleotides, including 8-oxo-dGTP, 8-oxoGTP, 8-oxo-dGDP and 8-oxoGDP, to their monophosphates, thereby preventing the misincorporation of 8-oxoG into DNA and RNA [4, 5]. The lack of the *mutT* gene cause significant increases in the spontaneous mutation frequency (up to 100 to 10,000 times the level in wild-type cells) [7]. Mammalian cells possess more elaborate mechanisms than bacteria to eliminate these oxidized nucleotides. Several counterparts of MutT protein, such as MTH1 (NUDT1), MTH2 (NUDT15), NUDT5, and MTH3 (NUDT18), have been identified. MTH1 can degrade 8-oxo-dGTP, 8-oxoGTP and 2-OH-dATP, but it hardly acts on 8-oxo-dGDP and 8-oxoGDP [8, 9]. In this regard, MTH2 is notable, since it exhibits the same substrate specificity as MutT despite having a low intrinsic activity [10]. MTH3, on the other hand, acts on 8-oxo-dGDP and 8-oxoGDP but not on 8-oxo-dGTP or 8-oxoGTP [11]. NUDT5 has potent activity to cleave ADP-sugars and also degrade 8-oxo-dGDP under alkaline conditions [12, 13]. Given these results, the MutT-related proteins may play significant roles in preventing replicational and transcriptional errors in mammalian cells.

It has been shown that the expression of MTH1 messenger RNA in renal-cell carcinoma is significantly higher than in adjacent non-tumorous kidney [14]. Similar observations have been made with other types of cancer, including breast tumors [15], brain tumors [16], non-small-cell lung carcinomas [17] and gastric cancer [18]. More recently, Akiyama *et al* showed that overexpression of MTH1 is associated with a poor prognosis in esophageal squamous cell carcinoma [19]. These results suggested that MTH1 activity is required for reproduction in cancer cells, whereas it is non-essential for the growth of normal cells. It was shown, furthermore, that small molecules that make complexes with MTH1 are effective for selectively inhibiting growth of cancer cells [20, 21]. However, no studies have explored the expression of other MutT-related proteins in human cancer, and the clinical relevance as well as disease outcome of these proteins in cancer patients are unclear. As colorectal cancer (CRC) is one of the most common malignant tumors [22] and clinical samples are relatively easy to come by, we focused on CRC in our research.

In the present study, we firstly examined the expression of MTH1, MTH2, MTH3 and NUDT5 in CRC cell lines and CRC specimens. We then evaluated the association of MTH1, MTH2, MTH3 and NUDT5 expression with clinicopathological features and the patient survivals. Finally, we depleted these MutT-related proteins using siRNA to investigate their effects on CRC cell proliferation.

## RESULTS

### Expression of *MutT-related genes* in human CRC cell lines

**Figure 1:**
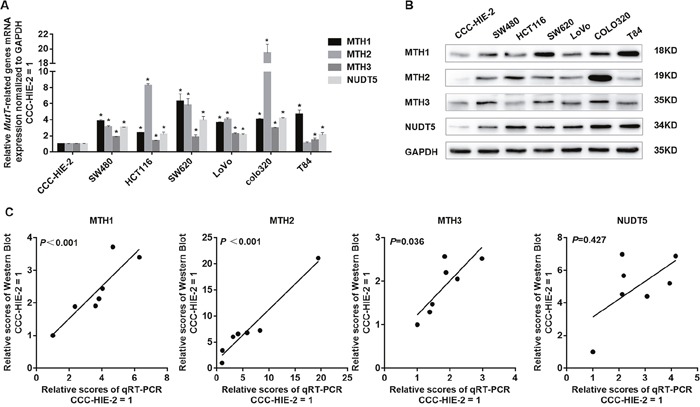
Expression of *MutT-related genes* in human colorectal cell lines **(A)** Normalized levels of mRNA for *MTH1, MTH2, MTH3* and *NUDT5* in six CRC cell lines and normal intestinal mucous cell line CCC-HIE-2, evaluated by qRT-PCR (Student's *t*-test, ^*^P<0. 05, compared with CCC-HIE-2). **(B)** Western blotting of MTH1, MTH2, MTH3 and NUDT5 proteins in these colorectal cell lines and CCC-HIE-2. **(C)** Correlation between amounts of mRNA and protein in each cell line. *P* values were determined by Spearman's rank correlation coefficient.

A previous study showed that levels of *MTH1* and *MTH2* mRNA are elevated in many cancer cells, similar to *MTH1* protein [20]. To assess the *MutT-related gene* expression as a whole, we determined levels of mRNA for *MTH1, MTH2, MTH3* and *NUDT5* in six kinds of human CRC cell lines (HCT116, SW480, SW620, LoVo, COLO320 and T84) and the human normal intestinal mucous cell line CCC-HIE-2 by performing quantitative real-time PCR (qRT-PCR). The normalized values for *MTH1, MTH2, MTH3* and *NUDT5* mRNA in the six CRC cell lines were significantly higher than those of CCC-HIE-2, except for MTH2 in T84 (Student's *t*-test, P<0.05, Figure 1A). We then performed Western blotting to detect *MutT-related* proteins in these cell lines. The normalized *MTH1, MTH2, MTH3* and *NUDT5* protein levels in the six CRC cell lines were also significantly higher than those of CCC-HIE-2, except for MTH3 in HCT116 (Student's *t*-test, *P*<0. 05, Figure 1B and Supplementary Figure 1). These results demonstrated that the expression of *MutT-related genes* was significantly upregulated in CRC cell lines. Since *MTH1, MTH2* and *MTH3* mRNA levels were well significantly associated with the protein levels, it seems that the expression of these three genes are primarily controlled at the transcriptional level (Spearman's rank correlation coefficient, *P*<0.05, Figure 1C). However, there was no obvious correlation between *NUDT5* mRNA and protein levels (*P*=0.427, Figure 1C), suggesting the expression of NUDT5 is regulated at both the transcriptional and post-transcriptional levels.

### High expression of MutT-related proteins and high levels of DNA and RNA oxidation in human CRC specimens

**Figure 2:**
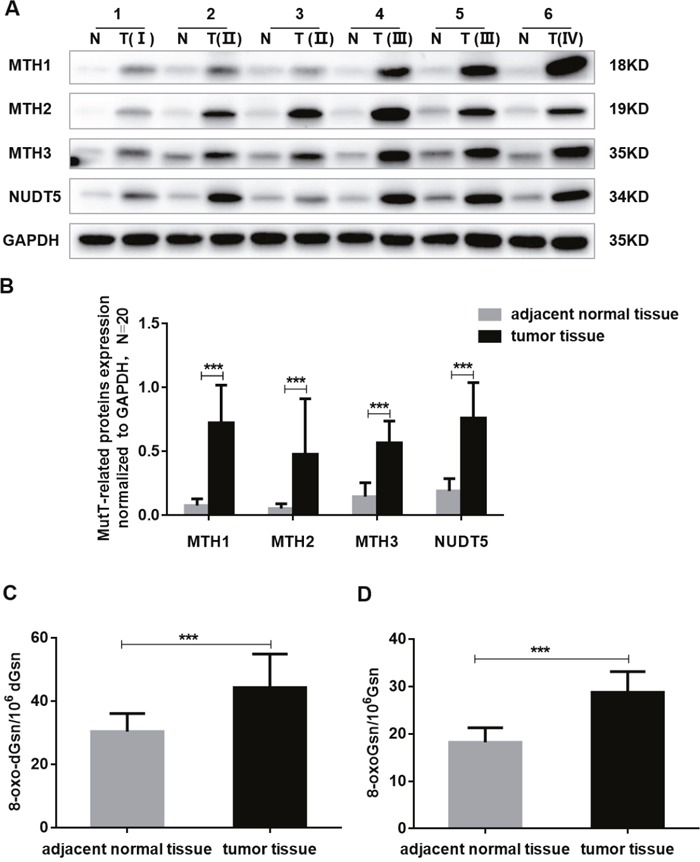
The expression of MutT-related proteins and the DNA and RNA oxidation levels in human CRC specimens **(A)** Amounts of MTH1, MTH2, MTH3 and NUDT5 proteins in six CRC tissue (T) and paired adjacent normal tissue (N) by Western blotting. **(B)** Quantification of MTH1, MTH2, MTH3 and NUDT5 proteins expression normalized against GAPDH in 20 paired CRC specimens. *P* values were calculated by Student's *t*-test, ^***^P<0.001. **(C-D)** The DNA and RNA oxidation levels of 20 paired CRC and adjacent normal tissues were examined by LC-MS/MS. *P* values were calculated by Student's *t*-test, ^***^P<0.001.

A Western blotting analysis of MutT-related proteins in 20 paired CRC samples and adjacent normal tissues showed that the expression of MTH1, MTH2, MTH3 and NUDT5 was significantly upregulated in tumor tissues. The mean expression, normalized against GAPDH, of MTH1, MTH2, MTH3 and NUDT5 in tumor tissues were 0.724±0.295, 0.478±0.435, 0.568±0.171 and 0.761±0.278 (mean±SD), respectively, which were significantly higher than the mean values of 0.077±0.052, 0.054±0.037, 0.147±0.109 and 0.191±0.096 for normal tissues, respectively. The overall expression of tumor tissues was about 3 to 9 fold that of normal tissues (Student's *t*-test, *P*<0. 001, Figure 2A-2B).

We compared the DNA and RNA oxidation levels of 20 cases of CRC and adjacent normal tissues using LC-MS/MS. The DNA and RNA oxidation levels of CRC were found to be significantly higher than those of normal tissues (Student's *t*-test, *P*<0. 001) (Figure 2C-2D).

**Figure 3:**
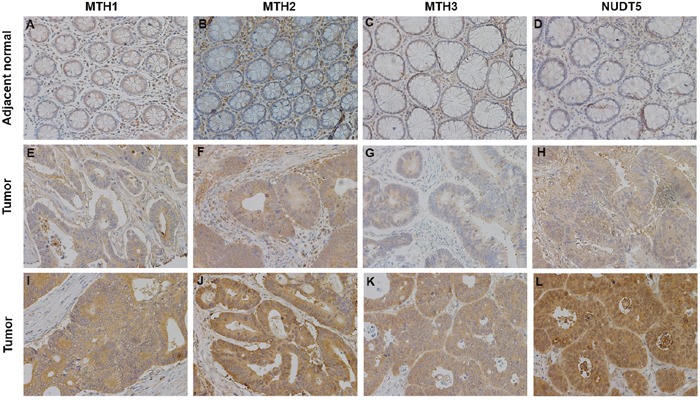
Representative immunohistochemical staining for MTH1, MTH2, MTH3 and NUDT5 expression in CRC specimens (X200) **(A-D)** Very low MTH1, MTH2, MTH3 and NUDT5 expression in adjacent normal tissues. **(E-H)** Low expression of MTH1, MTH2, MTH3 and NUDT5 in tumor tissues. **(I-L)** High expression of MTH1, MTH2, MTH3 and NUDT5 in tumor tissues.

Immunohistochemical analyses of tissue microarrays (TMAs) consisting of 87 paired CRC specimens verified that MutT-related proteins were overexpressed in tumor tissues. Positive immunostaining of MTH1, MTH2, MTH3 and NUDT5 was relatively weak in adjacent normal tissues (Figure 3). Tumor tissues showed different degrees of positive staining, including weak, moderate and strong. According to the intensity and proportion of immunostaining, relatively high expression of MTH1, MTH2, MTH3 and NUDT5 was observed in 54, 49, 45 and 42 of 87 CRC specimens, respectively. Representative immunohistochemical staining images for MutT-related proteins are shown in Figure 3.

### Clinicopathological significance of MutT-related proteins expression in human CRC

The clinicopathological factors analyzed in association with the levels of MutT-related proteins in CRC tissues by performing IHC of TMAs are summarized in Table 1. The expression of MTH1, MTH2, MTH3 and NUDT5 was positively significantly correlated to AJCC stage and N stage (Pearson’ *χ^2^* or Fisher's exact tests, *P*<0. 05).

**Table 1:** Relationship between clinicopathological parameters and expression of MutT-related proteins (n = 87)

	Total	MTH1		*P*	MTH2		*P*	MTH3		*P*	NUDT5		*P*
		Low	High		Low	High		Low	High		Low	High	
Age(years)													
<65	37	17	20		17	20		22	15		20	17	
≥65	50	16	34	0.185^a^	21	29	0.714^a^	20	30	0.073^a^	25	25	0.708^a^
Gender													
Male	45	16	29		20	25		21	24		24	21	
Female	42	17	25	0.636^a^	18	24	0.881^a^	21	21	0.756^a^	21	21	0.756^a^
Location													
Right	42	18	24		19	23		19	23		21	21	
others	45	15	30	0.695^a^	19	26	0.777^a^	23	22	0.584^a^	24	21	0.756^a^
Tumor size(cm)													
<5	37	11	26		13	24		14	23		15	22	
≥5	50	22	28	0.175^a^	25	25	0.167^a^	28	22	0.094^a^	30	20	0.073^a^
AJCC stage													
I+II	55	26	29		29	26		32	23		33	22	
III+IV	32	7	25	0.019^a*^	9	23	0.015^a*^	10	22	0.019^a*^	12	20	0.043^a*^
T stage													
T1+T2	12	8	4		4	8		7	5		7	5	
T3+T4	75	25	50	0.059^b^	34	41	0.436^a^	35	40	0.453^a^	38	37	0.622^a^
N stage													
N0	57	26	31		30	27		33	24		34	23	
N1+N2	30	7	23	0.042^a*^	8	22	0.020^a*^	9	21	0.013^a*^	11	19	0.041^a*^
M stage													
M0	84	33	51		37	47		41	43		44	40	
M1	3	0	3	0.285^b^	1	2	1.000^b^	1	2	1.000^b^	1	2	0.608^b^
Differentiation													
Well+Moderate	74	31	43		34	40		39	35		41	33	
Poor	13	2	11	0.119^b^	4	9	0.309^a^	3	10	0.071^b^	4	9	0.101^a^
Vascular invasion													
No	80	32	48		34	46		38	42		41	39	
Yes	7	1	6	0.245^b^	4	3	0.694^b^	4	3	0.707^b^	4	3	1.000^b^

^a^Chi-square test.

^b^Fisher's exact test.

^*^P<0.05.

Western blotting data of MutT-related proteins in 44 CRC specimens support the view that high expression of MTH1, MTH2, MTH3 and NUDT5 was significantly associated with the AJCC stage, T stage and N stage (Student's *t*-test, *P*<0. 05, Supplementary Table 1).

### Survival analyses and the prognostic significance of MutT-related proteins in human CRC patients

**Figure 4:**
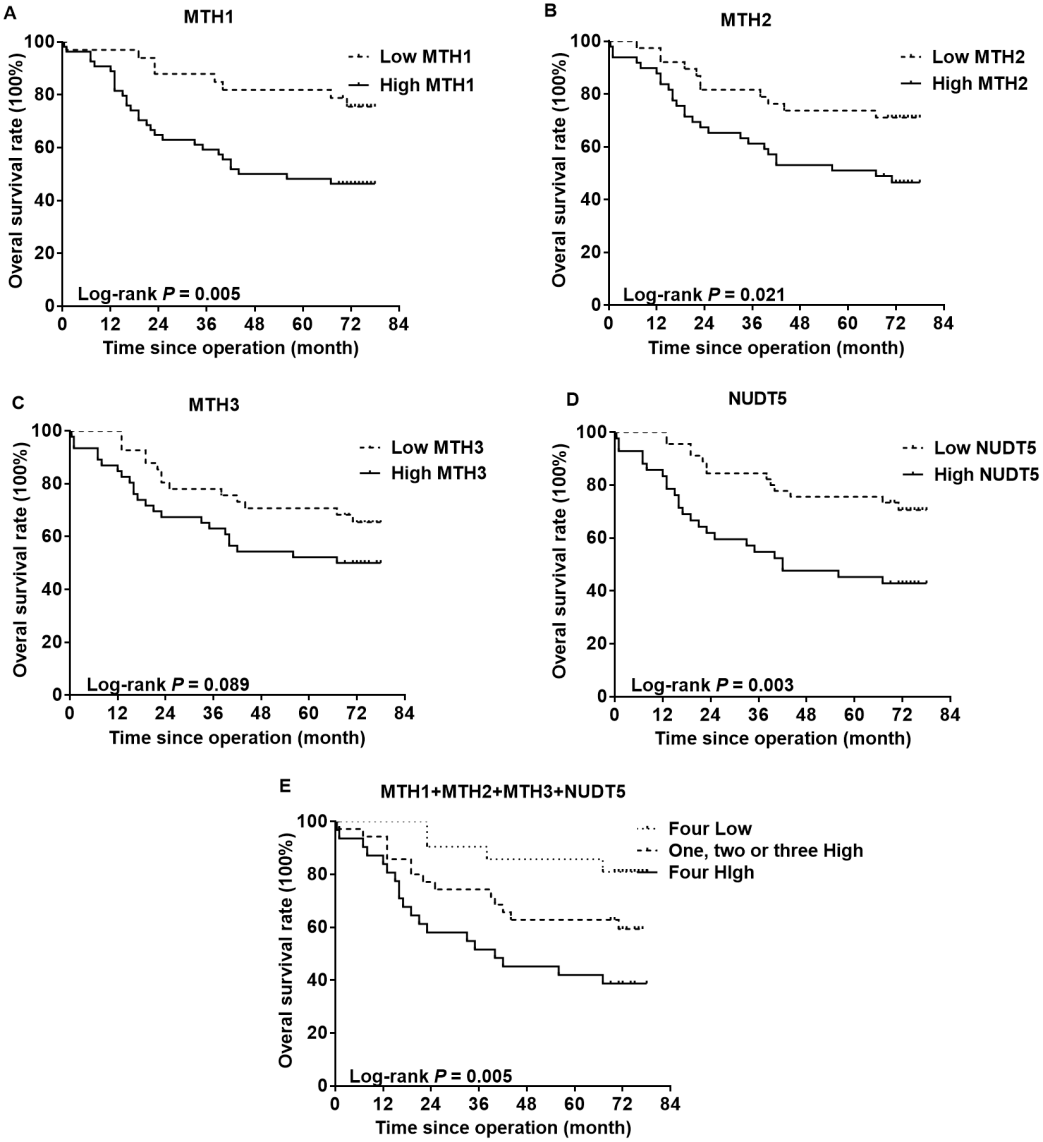
Kaplan-Meier curves for the overall survival rate of CRC patients The association of MTH1 **(A)**, MTH2 **(B)**, MTH3 **(C)** or NUDT5 **(D)** expression with the OS rate of CRC patients was evaluated by immunohistochemical staining of TMAs. **(E)** CRC patients exhibiting a high expression of all four types of proteins showed the lowest survival. *P* values were calculated by the log-rank test.

The association of MutT-related proteins with the OS rate of CRC patients was evaluated by performing a Kaplan-Meier analysis with a log-rank test of TMAs (Figure 4). CRC patients with high expression of either MTH1, MTH2 or NUDT5 had a significantly lower OS than those with a low expression (*P*=0.005, 0.021, 0.003, respectively). However, no such correlation was evident with MTH3 (*P*=0.089). After considering the situations in which these proteins are co-expressed, the samples were divided into three groups: the first group included tumors with low expression of all four proteins; the second group included tumors with high expression of one to three of the four proteins and the third group included tumors with high expression of all four proteins. Notably, the third group exhibited the lowest OS rate, followed by the second group (*P*=0.005).

As shown in Table 2, a univariate analysis with the Cox proportional hazards model revealed that a lower OS was significantly associated with the following characteristics: AJCC stage (*P*<0. 001), N stage (*P*=0.001), M stage (*P*=0.006), histological differentiation (*P*=0.003), MTH1 expression (*P*=0. 008), MTH2 expression (*P*=0.026) and NUDT5 expression (*P*=0. 005). Furthermore, a multivariate analysis indicated that a high NUDT5 expression was an independent and significant prognostic factor for the OS rate of CRC patients (hazard ratio [HR] 2.282; 95% confidence interval [CI] 1.152-4.517; *P*=0.018).

**Table 2:** Univariate and multivariate analysis of the overall survival

	Univariate analysis		Multivariate analysis	
	HR (95%CI)	*P*	HR (95%CI)	*P*
Age(years)				
<65	1			
≥65	1.178(0.617-2.249)	0.619		
Gender				
Male	1			
Female	0.707(0.367-1.364)	0.301		
Location				
Right	1			
Others	1.326(0.692-2.544)	0.395		
Tumor size (cm)				
<5	1			
≥5	2.088(0.870-5.008)	0.899		
AJCC stage				
I+II	1			1
III+IV	3.675(1.896-7.126)	<0.001^*^	3.320(1.700-6.481)	<0.001^*^
T stage				
T1 + T2	1			
T3 + T4	1.488(0.527-4.201)	0.453		
N stage				
N0	1			1
N1 + N2	3.099(1.617-5.940)	0.001^*^	0.894(0.077-10.418)	0.929
M stage				
M0	1		1	
M1	5.622(1.632-19.372)	0.006^*^	2.858(0.801-10.196)	0.106
Differentiation				
Well + Moderate	1		1	
Poor	3.142(1.472-6.706)	0.003^*^	1.542(0.645-3.687)	0.330
Vascular invasion				
No	1			
Yes	0.975(0.299-3.176)	0.967		
MTH1				
Low	1		1	
High	2.908(1.327-6.373)	0.008^*^	1.680(0.687-4.109)	0.255
MTH2				
Low	1		1	
High	2.233(1.102-4.522)	0.026^*^	0.523(0.130-2.103)	0.361
MTH3				
Low	1			
High	1.761(0.906-3.424)	0.095		
NUDT5				
Low	1		1	
High	2.639(1.342-5.191)	0.005^*^	2.282(1.152-4.517)	0.018^*^

^*^P<0.05.

### Knockdown of MutT-related proteins in CRC cells

**Figure 5:**
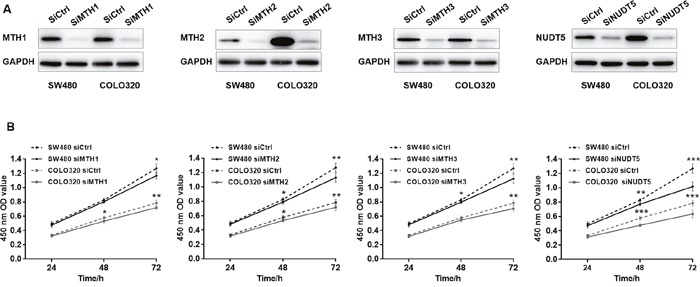
Knockdown of MutT-related proteins reduces the proliferation rate in SW480 and COLO320 cells **(A)** Western Blotting analysis of MTH1, MTH2, MTH3 and NUDT5 in knockdown and control cells. **(B)** Effects of knockdown of MutT-related proteins on cell proliferation as detected by CCK-8 assay. *P* values were calculated by Student's *t*-test, ^*^P<0.05, ^**^P<0.01, ^***^P<0.001.

Since the expression of MutT-related proteins was strongly increased in CRC tissues compared with adjacent normal tissues, we explored the potential effects of MutT-related proteins on CRC cell proliferation. Human CRC-derived cell lines, SW480 and COLO320, were transfected with siMTH1, siMTH2, siMTH3, siNUDT5 or control siRNA (siCtrl) to knockdown the expression of each of these proteins. The expression of each of the MutT-related proteins was considerably reduced, as shown by Western blotting (Figure 5A). Under these conditions, we performed a CCK-8 assay, which revealed that both SW480 and COLO320 cells transfected with siMTH1, siMTH2, siMTH3 or siNUDT5 showed a significantly decreased proliferative ability compared to cells transfected with control siRNA (Student's *t*-test, *P*<0. 05, Figure 5B).

## DISCUSSION

8-oxo-dGTP is produced by either the oxidation of dGTP or the phosphorylation of 8-oxo-dGDP, the latter of which is formed by the oxidation of dGDP. 8-oxo-dGTP thus formed can be incorporated into DNA and causes mispair with adenine during DNA replication, leading to the induction of A:T to C:G transversion mutations [4]. In the RNA precursor pool, 8-oxoGTP is formed in a similar manner and incorporated into RNA, resulting in transcriptional and translational errors. This causes the formation of abnormal proteins [5]. In *E. coli*, MutT protein has a potent activity to degrade 8-oxoGua-containing nucleotides to prevent such outcomes. We examined the roles of four types of MutT-related proteins, MTH1, MTH2, MTH3 and NUDT5, in mammalian cells.

Previous studies showed that the MTH1 expression is considerably higher in cancer cells than in normal cells [14–19]. In this study, we found for the first time that the MTH2, MTH3 and NUDT5 expression was prevalently upregulated in CRC cell lines (Figure 1) and CRC tissues (Figure 2). Furthermore, high expression of MutT-related proteins was significantly associated with AJCC stage and lymph node metastasis of CRC specimens (Table 1), and a Kaplan-Meier analysis showed that CRC patients with high expression of MTH1, MTH2 or NUDT5 had a significantly lower OS after surgical resection than those with low expression (Figure 4). Notably, CRC patients exhibiting a high expression of all four types of proteins showed the lowest survival among expression patterns. In addition, a Cox regression model analysis indicated that NUDT5 was a new and independent predictor for the prognosis of CRC patients.

The proteins MutT, MTH1, MTH2, MTH3 and NUDT5 all belong to the NUDIX hydrolase superfamily, most of which carry the 23-residue MutT-related sequence and possess catalytic activities to hydrolyze nucleoside diphosphatates linked to other moieties, X (NUDIX), with considerable substrate diversity [11]. It was shown that the expression of cDNA for MTH1, MTH2 or NUDT5 significantly reduced the elevated level of spontaneous mutation frequency in *mutT*-deficient *E. coli* [12, 23, 24]. Kamiya *et al* observed that knockdown of MTH1, MTH2 or NUDT5 in human 293T cells increased A:T to C:G substitution mutations induced by 8-oxo-dGTP. Since the increase in the induced mutation frequency was more evident in the triple-knockdown cells, it was suggested that these proteins act as a defense against the mutagenesis induced by oxidized dGTP [25]. We further showed that the expression of MTH1 and NUDT5, but not MTH2, in *E.coli mutT* deficient cells almost completely suppressed the increased production of erroneous proteins [26]. These results indicate that these MutT-related proteins help maintain the high fidelity of DNA replication and transcription under oxidative stress. However, few studies have so far explored the functions of MTH3.

Gad *et al* observed that knockdown of MTH1 protein in several cancer cells caused DNA damage and reduced the clonogenic survival and viability, implying that MTH1 is required for the cancer survival [20]. In addition, it was reported that the MTH1 expression was elevated in many types of human tumors and that MTH1 overexpression was correlated with the tumor pathological stage and a poor prognosis [14–19]. Given that cancers have dysfunction redox regulation and increased ROS tension, upregulated MTH1 expression can be useful for sanitizing the oxidized dNTP pool to prevent its incorporation into DNA, thus promoting the cancer survival and progression and contributing to the poor prognosis of cancer patients [19, 20]. In this study, we showed that the DNA and RNA oxidation levels of CRC were indeed significantly higher than those of adjacent normal tissues. MTH2, MTH3 and NUDT5 are also able to degrade oxidized nucleotides in order to ensure accurate replication and transcription. We were curious as to the potential relationship between elevated MTH2, MTH3 and NUDT5 expression in CRC tissues and cancer progression. In our present study using a CCK-8 assay, we showed that knockdown of MTH1˴MTH2˴MTH3 or NUDT5 in SW480 and COLO320 cells reduced the rates of cell proliferation, implicating that MutT-related proteins at large play roles in CRC cell proliferation.

Yu *et al* found that MTH2 directly interacted with proliferating cell nuclear antigen (PCNA) and that the knockdown of MTH2 significantly promoted PCNA degradation, suggesting PCNA-MTH2 complex may protect PCNA from degradation [27]. Furthermore, they showed that the knockdown of MTH2 inhibited DNA synthesis and caused an obvious G_1_-phage arrest in A549 cells, which was partly associated with the degradation of PCNA. PCNA is a useful proliferation marker, and its expression and distribution correlate with the cell proliferation rate and DNA synthesis [28]. Therefore, in addition to sanitizing oxidized nucleotides, upregulated MTH2 expression in CRC tissues may enhance PCNA stability to promote cell proliferation and tumor growth. Conversely, Carter *et al* observed that siRNA knockdown of MHT2 had no effect on 8-oxo-dGTP incorporation into DNA and cell cycle progression as well as the clonogenic survival in U2OS cells [29].

In the present study, we provided evidence that NUDT5 was a new prognostic factor not MTH1 for the OS according to multivariate analysis. Our previous study showed that knockdown of NUDT5 in Hela cells inhibited cell viability due to the G_1_-phase delay, via the upregulation of p53, p16 and Rb and downregulation of p-Rb [30]. Remarkably, in addition to degrading oxidized nucleotides, NUDT5 has a potent ability to cleave ADP ribose [13]. More recently, Wright *et al* proposed a mechanism for ATP production in the nuclei of breast cancer cells exposed to hormones. They showed that, in the presence of pyrophosphate, ADP-ribose is used by NUDT5 to generate nuclear ATP, which is indispensable to hormone-induced chromatin remodeling, transcriptional regulation, and cell proliferation [31]. Taken together, these findings suggest that an elevated level of NUDT5 expression may promote cancer progression in several ways.

Based on the observations that MTH1 prevented 8-oxo-dGTP incorporation and was required for the efficient survival of cancer cells, MTH1 was proposed as an anticancer target. Since the first-in-class MTH1 inhibitors TH588 and TH287 were confirmed to induce oxidative DNA damage, cytotoxicity and suppressive response to xenograft tumors [20], the addition of MTH1 inhibitors or knockdown of MTH1 via shRNA, siRNA, or CRISPR has been performed in a variety of cancer cells in order to evaluate their therapeutic utility [32–35]. Our present finding that MTH2, MTH3 and NUDT5 expression was associated with CRC progression and the prognosis, suggested that these proteins, in addition to MTH1, may be potential therapeutic targets for CRC.

It should be noted that there are some limitations of our present study. Firstly, the effects of MutT-related proteins knockdown are not yet investigated *in vivo*. Secondly, the expression of MutT-related proteins in other types of cancer, such as lung cancer, liver cancer, breast cancer and so on, is being considered. Thirdly, thorough investigations are required to elucidate the role of MutT-related proteins in CRC progression and their potential as anticancer targets.

## MATERIALS AND METHODS

### Cell lines and cell culture

The human CRC cell lines SW480, COLO320, T84 were purchased from the American Type Culture Collection (ATCC; Rockville, MD, USA). The human normal intestinal mucous cell line CCC-HIE-2 and other CRC cell lines SW620, LoVo and HCT116 were obtained from the Type Culture Collection of the Chinese Academy of Medical Sciences (Beijing, China). The cell lines were maintained at 37°C in a 5% humidified CO_2_ atmosphere. CCC-HIE-2 cells were cultured in DMEM supplemented with 20% FBS and 2ng/ml EGF (Solarbio, Beijing, China). HCT116, LoVo and other CRC cell lines were cultured in IMDM, F12K and RPMI-1640, respectively, with 10% FBS.

### Patients and specimens

Specimens of colorectal cancer tissue and corresponding adjacent normal tissue (>10 cm away from the cancer tissue) were collected from 44 CRC patients, who had been diagnosed and received surgery without preoperative radiotherapy or chemotherapy treatment at the First Affiliated Hospital of Jiamusi University, Heilongjiang, China, from 2014-2015. The resected tissues were immediately stored in liquid nitrogen prior to protein extraction. The detailed patient demographic information is shown in Supplementary Table 1. This study was approved by the Institutional Review Boards of the First Affiliated Hospital of Jiamusi University.

Four tissue microarrays (TMAs) containing 87 paired CRC specimens were purchased from Xin Chao Company (Shanghai Outdo Biotech Co., Ltd., China). The patients involved underwent operation between January 2009 and October 2009, and had been followed up until July 2015. The median survival time of the patients involved was 55.85 months (range, 0.4-78 months).

### Quantitative reverse transcription polymerase chain reaction

The total RNA was isolated from seven kinds of cell lines using TRIzol reagent (Thermo Fisher Scientific, Boston, MA, USA), and the cDNA was synthesized from 4 μg RNA in total volume of 40 μl using TransScript One-Step gDNA Removal and cDNA Synthesis SuperMix (TransGen Biotech, Beijing, China). Quantitative polymerase chain reaction (qPCR) was performed using KAPA SYBR® FAST Universal qPCR Kits (Kapa Biosystems, Boston, MA, USA). Each reaction was performed in triplicate. The relative mRNA expression of *MTH1, MTH2, MTH3* and *NUDT5* genes was normalized to that of *GAPDH*. The primer sequences used for qPCR were as follows: *MTH1*, forward 5′-CTCAGCGAGTTCTCCTGG-3′ and reverse 5′-GGAGTGGAAACCAGTAGCTGTC-3′ [20]; *MTH2*, forward 5′-GAAAGGAGAAGTGGATGTGAC-3′ and reverse 5′-GGAACCCACTCCCAACTTTC-3′ [25]; *MTH3*, forward 5′-ACTTGCCTGTCACTGCCTGT-3′ and reverse 5′-CCAAGTGGTGCAGGGTCAGA-3′; *NUDT5*, forward 5′-GTTCTCCAGCGGTCTGTATG-3′ and reverse 5′-CTTCGGCCTTGCGTTTTCG-3′ [25]; *GAPDH*, forward 5′-CCTCTCCAGAACATCATCC-3′ and reverse 5′-GTGTCGCTGTTGAAGTCAG-3′.

### Western blotting

The total proteins were extracted from cell lines or frozen tissues using RIPA lysis buffer (Solarbio) containing 1X PMSF (Solarbio) and 1X Protease/Phosphatase Inhibitor Cocktail (Cell Signaling Technology, Beverly, MA, USA) and the protein concentration was measured using a Pierce BCA Protein Assay Kit (Thermo Fisher Scientific). Then the proteins were diluted to 2 μg/μl by adding lysis buffer and 6X loading buffer and denatured at 100°C for 5min. 20 μg proteins for detecting MTH1 and NUDT5 or 40 μg proteins for detecting MTH2 and MTH3 were subjected to 12%SDS and Western blotting according to the standard procedure. The following antibodies were used: anti-MTH1 rabbit polyclonal antibody (ab187531; abcam, Cambridge, UK; 1:1000 dilution), anti-MTH2 rabbit polyclonal antibody (A8368; ABclonal, Wuhan, Hubei, China; 1:1000 dilution), anti-MTH3 mouse monoclonal antibody (ab123903; abcam; 1:2000 dilution), anti-NUDT5 rabbit monoclonal antibody (ab129172; abcam; 1:2000 dilution), and anti-GAPDH mouse antibody (TA-08; ZSGB-BIO, Beijing, China; 1:2000 dilution). The integrated density values of bands were determined by the Image J software program (NIH). Of note, the protein expression of MTH1, MTH2, MTH3 and NUDT5 was normalized to that of GAPDH. The experiment was repeated three times.

### Determination of DNA and RNA oxidation levels using LC-MS/MS

The method established in our laboratory was used [36]. Briefly, RNA and DNA were extracted from frozen CRC tissues and adjacent normal tissues followed by nuclease P1 (Wako, Osaka, Japan) and alkaline phosphatase (New England Biolabs Inc., Beverly, USA) digestion. We used Agilent-6490-Triple-Quad-LC/MS to detect 8-oxoGsn and Gsn in RNA and 8-oxo-dGsn and dGsn in DNA. The ratios of 8-oxoGsn/10^6^ Gsn for RNA and of 8-oxo-dGsn/10^6^ dGsn for DNA were calculated.

### Immunohistochemistry

Four tissue microarrays were de-waxed using xylene, gradually dehydrated with a series of graded ethanol washes and then washed with PBS, followed by citric acid buffer (pH 6.0) microwave antigen retrieval for 45 min. After being treated with 3%H_2_O_2_ for 15 min and blocked with 10% normal goat serum (ZSGB-BIO), the 4 sections were respectively incubated overnight in the dark at 4°C with the following antibodies: anti-MTH1 antibody (1:400 dilution), anti-MTH2 antibody (1:200 dilution), anti-MTH3 antibody (1:600 dilution) and anti-NUDT5 antibody (1:1000 dilution). These sections were then treated with a Polink-1 HRP DAB Detection System (ZSGB BIO) for 20 min and visualized by a DAB kit (ZSGB-BIO). Finally, all sections were counterstained with hematoxylin (ZSGB-BIO). Another CRC specimen was incubated with normal goat serum instead of the primary antigen as a negative control. Images were observed and photographed using a Nikon Eclipse 80i microscope (Nikon, Tokyo, Japan).

MTH1, MTH2, MTH3, and NUDT5 immunoreactivity was divided into low expression and high expression according to the intensity and proportion of immunostaining of the specimens. The staining intensity was scored as 0 (negative), 1 (weak), 2 (moderate) and 3 (strong). The staining proportion of the positive cells was scored as 0 (0%), 1 (1-25%), 2 (26-50%), 3 (51-75%) and 4 (76-100%). The final score was obtained by multiplying the staining intensity by the staining proportion, with 0-6 considered as low expressionand 7-12 high expression.

### RNA interference and CCK-8 assay

The small interfering RNA target sequences of MutT-related genes were as follows: *MTH1*, 5′-CGACGACAGCTACTGGTTT-3′; *MTH2*, 5′-GGAT GTGACTCATGATTCA-3′; *MTH3*, 5′-CCAAGAGCT ACCCTGTGAT-3′; *NUDT5*, 5′-CATGGATCCTACTG GTAAA-3′. The specific siRNAs were synthesized by RiboBio Co., Ltd (Guangzhou, China).

Cells (1×10^4^) were seeded in 96-well plates and transfected with control siRNA (siCtrl), siMTH1, siMTH2, siMTH3, or siNUDT5, respectively. After incubation for 24, 48 and 72h, 100 μl complete medium containing 10 μl CCK-8 reagent (Beyotime, Jiangsu, China) was added to each well and was incubated at 37°C for 1h. The absorbance of each well at 450 nm was measured using a Tecan Genios microplate reader (TECAN, Grodig, Austria). Six wells were assessed for each group, and the results were presented as mean ± standard deviation (SD).

### Statistical analyses

All data analyses involved in this study were performed using the SPSS statistics software program, version 19. Student's *t*-test was used to compare the means from two divided groups. Spearman's rank correlation coefficient was used to evaluate the correlation between the expression of mRNA and protein. The association between MutT-related proteins and clinicopathological features was analyzed by Pearson’ χ*^2^* or Fisher's exact tests, when appropriate. A Kaplan-Meier analysis was used to calculate the overall survival (OS) and a log-rank test was used to compare the OS between two groups. Cox proportional hazards models were used to determine the effects of CRC clinicopathological variables and MutT-related proteins expression on the survival and prognosis of CRC patients. Variables with a significant *P* value in the univariate analysis were incorporated into subsequent multivariate analysis using a backward (ward) method. In this study, *P* value < 0.05 was considered to indicate statistical significance.

## SUPPLEMENTARY MATERIALS FIGURE AND TABLE


