# Supplementary material for: MutT-related proteins are novel progression and prognostic markers for colorectal cancer
Source: Oncotarget. 2017 Nov 11;8(62):105714–26. doi: 10.18632/oncotarget.22393 (PMC5739673; doi:10.18632/oncotarget.22393)
Supplement: Supplementary file 1 [file oncotarget-08-105714-s001.pdf]

MutT-related proteins are novel progression and prognostic markers for colorectal cancer

SUPPLEMENTARY MATERIALS

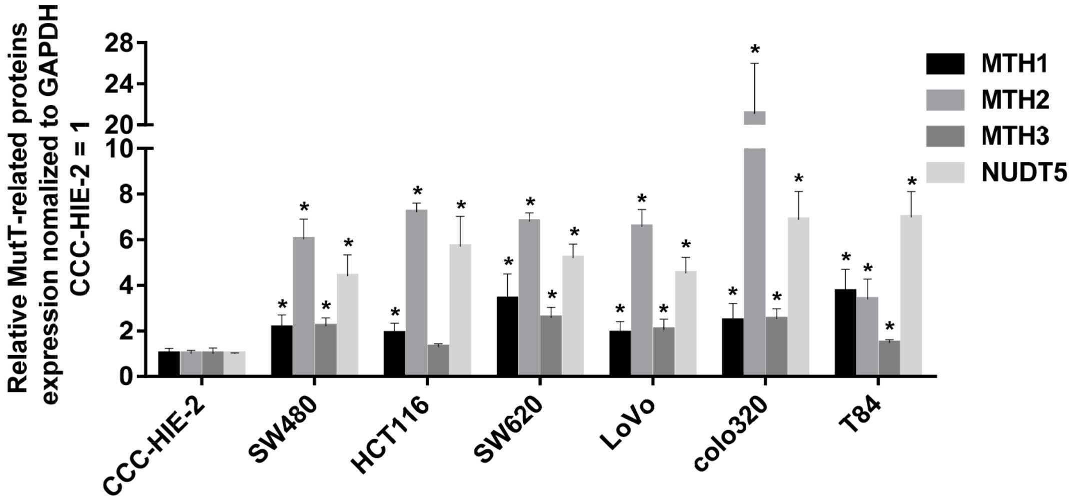

Supplementary Figure 1: Normalized MTH1, MTH2, MTH3 and NUDT5 protein levels in six CRC cell lines and normal intestinal mucous cell line CCC-HIE-2, obtained from Western blotting (Student's *t*-test, $^{*}P$ <0. 05, compared with CCC-HIE-2).

Supplementary Table 1: Relationship between clinicopathological parameters and expression of MutT-related proteins (n = 44)

Parameters	Total	MTH1		MTH2		MTH3		NUDT5	
		Mean±SD	P	Mean±SD	P	Mean±SD	P	Mean±SD	P
Age (years)									
<65	22	0.56±0.39		0.41±0.29		0.47±0.19		0.60±0.25	
≥65	22	0.70±0.40	0.173	0.58±0.43	0.134	0.50±0.18	0.603	0.72±0.35	0.190
Gender									
Male	21	0.65±0.39		0.47±0.33		0.45±0.18		0.66±0.36	
Female	23	0.60±0.29	0.617	0.52±0.41	0.682	0.51±0.18	0.288	0.66±0.25	0.960
Location									
Colon	23	0.60±0.34		0.48±0.34		0.47±0.18		0.63±0.32	
Rectum	21	0.66±0.35	0.540	0.52±0.41	0.72	0.50±0.19	0.587	0.69±0.29	0.471
Tumor size(cm)									
<5	26	0.59±0.35		0.50±0.42		0.45±0.17		0.59±0.25	
≥5	18	0.67±0.34	0.447	0.49±0.30	0.896	0.54±0.19	0.114	0.76±0.36	0.069
AJCC stage									
I+II	20	0.42±0.24		0.36±0.30		0.38±0.14		0.52±0.23	
III+IV	24	0.79±0.32	<0.001*	0.61±0.39	0.026*	0.57±0.17	<0.001*	0.78±0.32	0.004*
T stage									
T1+T2	8	0.30±0.10		0.23±0.10		0.35±0.09		0.44±0.08	
T3+T4	36	0.70±0.33	0.002*	0.56±0.38	0.024*	0.51±0.18	0.018*	0.71±0.31	0.020*
N stage									
N0	20	0.42±0.24		0.36±0.30		0.38±0.14		0.52±0.23	
N1+N2	24	0.79±0.32	<0.001*	0.61±0.39	0.026*	0.57±0.17	<0.001*	0.78±0.32	0.004*
M stage									
M0	39	0.61±0.35		0.47±0.36		0.49±0.19		0.66±0.32	
M1	5	0.74±0.27	0.451	0.70±0.45	0.204	0.46±0.16	0.769	0.69±0.12	0.821
Differentation									
Well+moderate	37	0.63±0.34		0.52±0.39		0.48±0.19		0.65±0.25	
Poor	7	0.61±0.40	0.921	0.37±0.21	0.318	0.48±0.18	0.964	0.72±0.52	0.572

SD, stardare deviation.
*P<0.05.
